# Communication: Demonstration of a 20 ps X-ray switch based on a photoacoustic transducer

**DOI:** 10.1063/1.4993730

**Published:** 2017-10-06

**Authors:** A. Jarnac, Xiaocui Wang, Å. U. J. Bengtsson, J. C. Ekström, H. Enquist, A. Jurgilaitis, D. Kroon, A. I. H. Persson, V.-T. Pham, C. M. Tu, J. Larsson

**Affiliations:** 1 MAX IV Laboratory, Lund University, P.O. Box 118, SE-221 00 Lund, Sweden; 2 Department of Physics, Lund University, P.O. Box 118, SE-221 00 Lund, Sweden

## Abstract

We have studied an X-ray switch based on a gold coated indium antimonide crystal using time-resolved X-ray diffraction and demonstrated that the switch could reduce the pulse duration of a 100 ps X-ray pulse down to 20 ps with a peak reflectivity of 8%. We have used a dynamical diffraction code to predict the performance of the switch, which was then confirmed experimentally. The experiment was carried out at the FemtoMAX beamline at the short-pulse facility of the MAX IV laboratory. The performance and limitation of the switch are discussed in terms of acoustic transport properties between the two materials and the electron transport properties of gold.

## INTRODUCTION

The study of structural dynamics using 3rd generation storage rings has been a rich research field for two decades. Electron storage rings provide unprecedented brilliance, stability, and tunability. A drawback is that these facilities rarely deliver pulses shorter than 100 ps. It is however possible to study ultrafast structural dynamics using the radiation from electron storage rings. Three methods that provide ultrashort electron bunches and therefore ultrafast X-ray pulses are low-alpha mode,^1,2^ femtoslicing,^3^ and the upcoming Variable pulse length Storage Ring upgrade of BESSY II (BESSY VSR).^4^ Low-alpha mode operation involves lowering the bunch charge and optimizing the machine lattice in order to obtain pulses as short as 5 ps. This is however done at the expense of lowering the X-ray flux for all users by about two orders of magnitude. Therefore, this mode of operation is only available a few weeks per year at a few facilities. Femtoslicing involves perturbing the electron trajectory using a femtosecond laser in a periodic magnetic field. This results in spatially or angularly separated beams which can be used for experiments. Difficulties with this method are that the overall flux is very low and the issue of temporal contrast can be quite severe for hard X-rays since some of the 100 ps light from the larger unperturbed electron bunch can be transmitted to the sample.^5^ Although methods have been devised to compensate for the halo,^6^ these have a limited applicability when a 2D detector is used. Finally, BESSY VSR involves upgrading the ring by installing superconducting radio frequency cavities. By using interferences produced by beating of harmonic and sub-harmonic radio frequencies, short electron bunches from 15 ps to 0.4 ps are expected to be produced at high current.

Another approach to obtain a high temporal resolution at synchrotron radiation facilities is the use of streak cameras.^7,8^ Streak cameras suffer from issues with low detection quantum efficiency, in the range of 1% and a limited temporal range around 100 times the temporal resolution which corresponds to 50 ps for very fast cameras.

An attractive method for obtaining ultrashort X-ray pulses is to switch the efficiency of a Bragg-reflection using a laser pulse. This has the advantage that it requires no changes to the accelerator or electron beam that could affect other beamlines at the facility. In 1999, a concept based on optical phonons was proposed,^9^ but it has so far not been possible to demonstrate it experimentally. A more successful approach has been to utilize zone-folded acoustic phonons in a superlattice.^10^ This switch generated a train of pulses; however, the concept was later improved by generating the strain wave in a thin metallic oxide film coated on top of an acoustically matched dielectric substrate (PicoSwitch^11,12^). The PicoSwitch has a few ps temporal response, a high temporal contrast but a diffraction efficiency of about 0.1%–0.5%. Another method is to use acoustic phonons in a substrate. X-ray scattering from a perfect lattice perturbed by acoustic phonons results in sidebands modulated by the phonon frequencies that are generated.^13,14^ It has also been shown that when the acoustic phonon spectrum is modulated, these sidebands can be enhanced or cancelled.^15^ Loether *et al.* proposed a method for switching X-rays based on modulating the acoustic phonon spectrum in a semiconductor by using an optoacoustic transducer.^16^ In that study, the X-ray reflectivity from photo-acoustic transducers was studied using a temporal resolution of 50 ps. With a better temporal resolution, it has been possible to observe enhanced/cancelled sidebands due to the modulated acoustic phonon spectrum.^17,18^ In particular, Persson *et al.* studied X-ray scattering from a nickel photo-acoustic transducer.

In the present work, we have designed a switch based on known material parameters and demonstrated that a gold/InSb switch can reduce the pulse duration of a 100 ps X-ray pulse to 20 ps with a peak reflectivity of 8%. The measurement is the first experiment to be carried out at the FemtoMAX beamline at the short-pulse facility^19^ of the MAX IV laboratory. The present work is part of the commissioning activities of this beamline.

## FEMTOMAX BEAMLINE

FemtoMAX is a beamline which has been built to produce 100 fs intense X-ray pulses for laser pump and X-ray probe experiments. FemtoMAX is anticipated to produce a stable beam, as opposed to SASE hard X-ray lasers, but with about as many photons per pulse as a storage ring, at a maximum repetition rate of 100 Hz. X-rays are presently generated by a temporary 2 m long undulator^20^ from MAX II that will be replaced by two 5 m long in-vacuum undulator sections from Hitachi/Neomax. X-rays were focused on the sample by a toroidal Rh-coated Si mirror. The spot size in this experiment was 0.15 mm × 0.09 mm. With the present undulator, the X-ray divergence is 120 *μ*rad. X-rays are monochromatized by a double crystal monochromator. In this study, we used an InSb monochromator with a resolution of 2 eV at an energy of 5 keV. During this beam time, the LINAC was run in the non-compressed mode, yielding an X-ray pulse duration of about 2 ps. To pump the sample, the beamline is equipped with a commercial Ti:Sapphire amplifier (KM Laboratories Red Wyvern) that can operate at a repetition rate up to 1 kHz and gives pulses of 40 fs (Fourier Transform Limited), 11 mJ at 800 nm. The repetition rate of the laser was reduced to match the one of the LINAC, which is presently restricted to 2 Hz. The laser pulses were synchronized to the electron bunches in the LINAC within an accuracy of 1 ps (rms). Further technical details about the beamline are reported elsewhere.^21^

## NUMERICAL SIMULATIONS

We simulated the performance of the switch which is composed of a 60 nm-thick (*d_Au_*) gold film deposited on an InSb (111) symmetrically cut crystal. We used the udkm1Dsim toolbox^22^ which models the response of 1D crystalline sample structures upon laser excitation and simulates the resulting transient X-ray diffraction efficiency. To evaluate the ultrashort laser heat deposition into the gold film and account for the electronic transport, we used the two-temperature model.^23^ The two temperature model can be implemented within the udkm1Dsim toolbox. This model treats the electrons and the lattice as two systems having their own temperature *T_e_* and *T_l_*, respectively. The electrons absorb the laser energy and then cool down by transferring energy to the lattice until the two systems reach equilibrium. The energy transfer between the two systems is given by

$$\begin{array}{c} A_{e}T_{e}\frac{\partial T_{e}\left(t,z\right)}{\partial t}=\frac{\partial }{\partial z}\left(k_{e}\frac{T_{e}}{T_{l}}\frac{\partial T_{e}\left(t,z\right)}{\partial z}\right)-G\times \left(T_{e}\left(t,z\right)-T_{l}\left(t,z\right)\right)+S\left(t,z\right), \end{array}$$

$$\begin{array}{c} C_{l}\frac{\partial T_{l}\left(t,z\right)}{\partial t}=G\times \left(T_{e}\left(t,z\right)-T_{l}\left(t,z\right)\right). \end{array}$$

All the parameters are given for gold, where *A_e_* = 67.6 J m^−3^ K^−2^ (Lin *et al.*^24^) is the electronic heat capacity, *k_e_* = 317 W m^−1^ K^−1^ (Haynes^25^) is the electronic thermal conductivity at 300 K, *C_l_* = 129 J kg^−1^ K^−1^ (Haynes^25^) is the lattice heat capacity, and *G* is the electron–phonon coupling factor. $S\left(t,z\right)=\frac{F_{abs,sim}}{\tau_{p}\zeta }e^{-z/\zeta }e^{-t^{2}/\tau_{p}^{2}}$ is the laser power initially deposited into the electron system, where *F_abs,sim_* = 4 mJ/cm^2^ is the absorbed laser fluence, *ζ* = 12.7 nm (Olmon *et al.*^26^) is the optical absorption depth, and *τ_p_* = 60 fs is the laser pulse duration at the sample. Due to the ballistic motion in gold, the hot electrons reach the interface between the two materials in a few hundred femtoseconds. This interface is a metal-semiconductor junction that forms a Schottky barrier.^27^ The height of the barrier *ϕ_B_* is given by $\phi_{B}=\phi_{Au}-\chi_{\text{InSb}}=0.7\;\text{eV}$, where $\phi_{Au}=5.3\;\text{eV}$ is the work function of Au^28^ and $\chi_{\text{InSb}}=4.6\;\text{eV}$ is the electron affinity of InSb.^29^ The electronic temperature in Au as calculated by the two temperature model reaches a maximum of 5400 K corresponding to an energy of 0.45 eV. This energy is insufficient for the hot electrons to cross the barrier. Thus, we assume that no electronic heat flows from Au into InSb. In the simulation, the lack of heat flow is accounted for by setting the electronic thermal conductivity (*k_e_*) of InSb to zero.

**FIG. 1.**
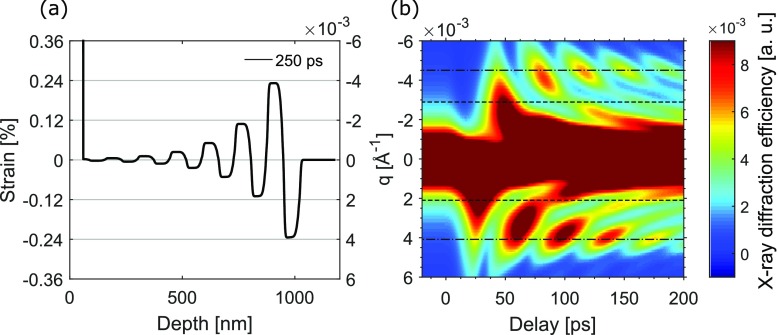
(a) Calculated strain wave as a function of depth in InSb at a delay of 250 ps for an absorbed laser fluence of *F_abs,sim_* = 4 mJ/cm^2^. (b) Simulated time-resolved X-ray diffraction efficiency from InSb as a function of phonon wave vector. The dot-dashed lines indicate the first enhanced phonon wave vectors. The dashed lines indicate the first cancelled phonon wave vectors.

Due to the fast electronic transport in Au, the heat is deposited in the electron system over the whole thickness of the Au film within a few picoseconds. Subsequently, the lattice is heated as the electron and lattice temperatures are equilibrated. This results in a stressed layer and the expansion of the Au film which launches a compressive strain wave into InSb. Additionally, two expansive strain waves (one originating from the Au surface and the other from the Au/InSb interface) counter-propagate in the Au layer.^30^ The reflections of these two waves at the interface, due to the acoustic mismatch, generate a succession of transmitted echoes in InSb. The strain wave in InSb, 250 ps after the laser illumination, can be seen in Fig. 1(a). The periodicity of the strain wave is given by the Au film thickness and corresponds to $T=2d_{Au}/c_{s,Au}∼38$ ps, where *c_s,Au_* = 3240 m/s is the longitudinal speed of sound in Au.^25^ As a result, phonon frequencies *ν_e,m_* with periods of m×T,  where *m* = 1, 3, 5,…, will be enhanced in InSb, whereas phonon frequencies *ν_c,n_* with periods of n×T, where *n *= 2, 4, 6,…, will be cancelled. The first enhancement (e) and cancellation (c) modes will thus be found for *ν_e,1_ *= 26 GHz and *ν_c,2_ *= 13 GHz, respectively. The photon energy can be set so that a particular acoustic phonon is required to fulfil the scattering condition^13,14^ $\vec{S_{0}}-\vec{S}=\vec{Q}\pm \vec{q}$, where $\vec{S_{0}}$ and $\vec{S}$ are the wave vectors of the incident and diffracted X-ray waves, respectively; $Q=\frac{2\pi }{d_{\text{InSb},111}}$ is the reciprocal lattice vector with *d_InSb,111_* being the (111) plane spacing; $q=2\pi \upsilon /c_{s,InSb}$ is the wave vector of the acoustic phonon, with *c_s,InSb_* = 3880 m/s is the longitudinal speed of sound in InSb (Slutsky and Garland^34^). In this experiment, with Q=1.68 Å^−1^, the central energy *E_0_* = 5018 eV and the Bragg angle *θ_B_* = 19.32°, the required phonon wave vector was $q_{e,1}∼\pm 4.18\times 10^{-3}{Å}^{-1}↔\Delta E_{e,1}∼\pm 13\;\text{eV}$ for enhanced and $q_{c,2}∼\pm 2.11\times 10^{-3}{Å}^{-1}↔\Delta E_{c,2}∼\pm 6\;\text{eV}\;$ for cancelled phonon frequencies. The relationship between $\vec{q}$ and ΔE can be calculated from $q/Q=\Delta E/E_{0}$. The manifestation of cancellations and enhancements of the phonons with these wave vectors are evident when calculating the dynamical X-ray diffraction efficiency induced by the time-dependent strain distribution. The calculated time-resolved X-ray diffraction efficiency as a function of the phonon wave vector and time is shown in Fig. 1(b). One can see that the enhanced/cancelled phonon wave vectors are in good agreement with the values calculated from the scattering equation.

## EXPERIMENTAL RESULTS

**FIG. 2.**
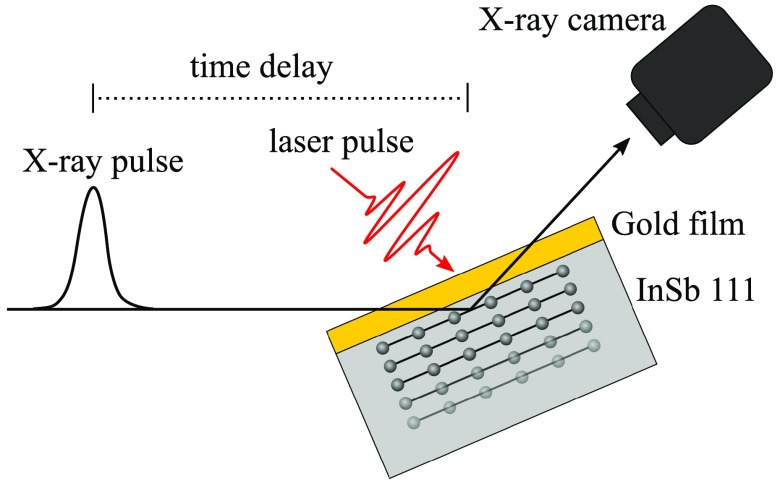
Sketch of the experimental setup. The switch—Au (60 nm)/InSb (111)—is illuminated by 60 fs laser pulses at 800 nm and a fluence of 33 mJ/cm^2^. The diffracted X-ray intensity from InSb (111) planes is recorded as a function of the time delay between the laser pulse and the X-ray pulse. The angle of incidence of the X-ray beam is *θ_B_* = 19.32° with respect to the sample surface. The angle of incidence of the laser beam is 50° with respect to the surface normal.

**FIG. 3.**
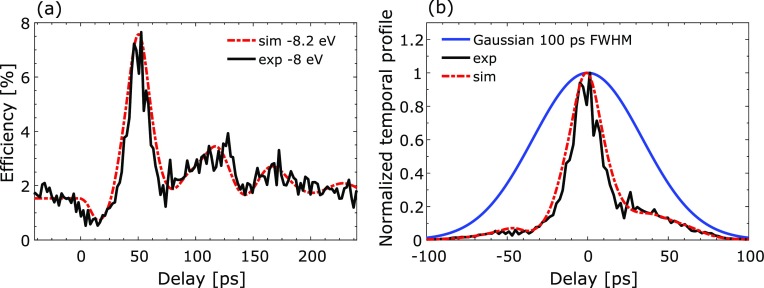
(a) Time-resolved X-ray diffraction efficiency from the switch: experimental data (solid black curve, $\Delta E=-8\;\text{eV}↔q=-2.68\times 10^{-3}\;{Å}^{-1}$) and simulated data (dot-dashed red curve, $\Delta E=-8.2\;\text{eV}\;↔q=-2.75\times 10^{-3}\;{Å}^{-1}$). (b) Normalized experimental data multiplied by a Gaussian pulse of 100 ps (FWHM) (solid blue curve). One can see the reduction in the pulse duration to 20 ps after the interaction with the switch.

We measured the time-resolved X-ray diffraction efficiency from the above-mentioned switch (Fig. 2). The switch was activated by an 800 nm, 60 fs laser pulse. At this wavelength, the optical absorption depth in gold [*ζ* = 12.7 nm (Ref. 26)] is smaller than the thickness of the film, which ensures that the laser energy is only deposited in the Au film. We set the laser spot size to 1.9 mm × 3.5 mm delivering an incident fluence of 33 ± 7 mJ/cm^2^, where the uncertainty arised partly from the non-Gaussian laser beam profile and the large beam size compared to the X-ray spot. The incidence angle of the laser beam on the switch was 50° with respect to the surface normal, and the laser beam had p-polarization. We measured a reflection coefficient of ∼ 0.9, which results in an absorbed fluence in the Au film of *F_abs,exp_ *= 3.3 mJ/cm^2^. The train of strain pulses arising from the film is transmitted and propagates into InSb in which we probed the time-resolved X-ray diffraction efficiency at an energy offset of $\Delta E=-8\;\text{eV}\;↔q=-2.68\times 10^{-3}$ Å^−1^. The experimental result is presented in Fig. 3(a) (solid black curve). The X-ray diffraction efficiency was acquired using a CCD camera (Andor iKon-L) with a time step of 2 ps for each time delay between the laser pulse and the X-ray pulse. The data are obtained from the total charge generated by each X-ray pulse with an average of 90 pulses per delay, corresponding to a total of 13 500 shots or 2 h of acquisition at the 2 Hz repetition rate of the LINAC. The data are normalized to the incident X-ray intensity and as a consequence can be read as the switch diffraction efficiency. The switch reflectivity exhibits a sharp peak for 20 ps [full width at half maximum (FWHM)], which reaches an efficiency of 8%. In order to illustrate the switch action at a synchrotron beamline, we multiply the time-dependent diffraction efficiency by a Gaussian profile with a pulse duration of 100 ps (FWHM) [Fig. 3(b)]. By selecting a suitable delay (peak of Gaussian pulse at 50 ps after the laser illumination), one can reduce the incoming 100 ps X-ray pulse to a 20 ps pulse with a temporal contrast of C_0_ = 9 and C_∞_ = 4, defined as $C_{0,\infty }=\frac{\eta_{max}-\eta_{0,\infty }}{\eta_{0,\infty }}$, where η is the diffraction efficiency before ($\eta_{0}$), after ($\eta_{\infty }$), and at the maximum ($\eta_{max}$) of the switch action.^11^ When comparing with the simulation (dot-dashed red curve in Fig. 3), an excellent agreement is found for an offset of $\Delta E=-8.2\;\text{eV}\;↔q=-2.75\times 10^{-3}$ Å^−1^ after the simulations have been convoluted in energy by a Lorentz function with a width of 2 eV (FWHM) and in time by a Gaussian function with a duration of 2 ps (FWHM) corresponding to the X-ray pulse duration.

## DISCUSSION

**FIG. 4.**
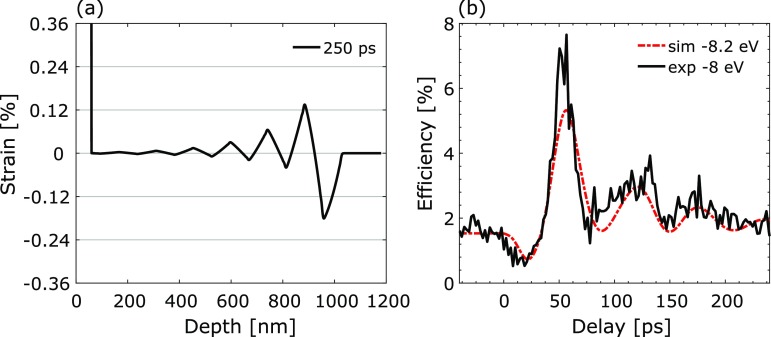
(a) Calculated strain wave as a function of depth in InSb at a delay of 250 ps for G = 1 × 10^16^ W m^−3^ K^−1^ corresponding to *t_eq_* ∼ 36 ps and *F_abs,sim_* = 4 mJ/cm^2^. (b) Comparison between experimental data and calculated time-resolved X-ray diffraction efficiency for the strain shown in (a).

The performance of the switch has many attractive properties, such as a high reflectivity and an intermediately high temporal resolution, which give a high throughput. The performance relies on the acoustic mismatch between the two materials and the electron transport properties of gold. It is also important that all parameters in the simulations are correct in order to be able to optimize the performance. The best fits for the simulation to the experiment are found for a minimum electron-phonon coupling factor in gold of *G* = 4 × 10^16^ W m^−3^ K^−1^. A further increase in *G* does not influence the shape of the simulated curve. The values of *G* in the literature span from 2 × 10^16^ to 1 × 10^17^ W m^−3^ K^−1^ depending on the electronic temperature,^24^ the experimental technique,^31^ or the nature of the substrate.^32^ The electron-phonon equilibration time is related to the coupling factor by the relation^33^ $t_{eq}=A_{e}T_{e}/G$, where *T_e_* = 5400 K is the maximum initial electronic temperature. For *G* = 4 × 10^16^ W m^−3^ K^−1^, this corresponds to *t_eq_* ∼ 9 ps which in our case is faster than the acoustic propagation time through the Au film $t_{ac}=d_{Au}/c_{s,Au}∼19$ ps. When *t_ac_* and *t_eq_* are similar in magnitude, the shape of the strain pulse train and thereby the time-resolved X-ray diffraction efficiency are very sensitive to small changes in the electron-phonon coupling factor. As can be seen in Fig. 4, the agreement between the simulation and the experimental data is significantly worse when an artificially low electron-phonon coupling factor of 1 × 10^16^ W m^−3^ K^−1^ (*t_eq_* ∼ 36 ps) was used.

It is straightforward to make the temporal response of the switch slower by increasing the gold film thickness and choosing an X-ray photon energy closer to matching the Bragg condition for the unperturbed lattice. It is however not equally straightforward to design a switch with a faster temporal response. One has to consider three conditions. First, if the propagation time of the acoustic wave *t_ac_* is faster than the electron-phonon equilibration time *t_eq_*, the stress does not have enough time to develop uniformly throughout the film, resulting in a lower strain with a different shape [see Fig. 4(a)]. This produces a lower X-ray diffraction efficiency and significantly reduces the efficiency and temporal contrast of the switch [see Fig. 4(b)]. As a consequence, the lower limit of the Au film thickness is set by $d_{Au}>t_{eq}\times c_{s,Au}$. In the frame of this study, we conclude a minimum film thickness of *d_min_* = 35 nm. Second, the film thickness imposes the condition for cancellation modes. A thinner film moves the first cancellation mode towards a higher phonon frequency and larger phonon wave vector, thereby shortening the response time of the switch. For *d_min_*, the first cancellation mode corresponds to $\Delta E_{c}∼\pm 13\;\text{eV}↔q_{c}∼\pm 4.18\times 10^{-3}{Å}^{-1}$ for *E_0_* = 5018 eV. Finally, the amount of strain strongly influences the intensity of the X-ray diffraction at the high energy offset required for destructive interference. According to our simulation, such a switch would give a temporal response of 15 ps at an energy offset of −15.2 eV and would require an absorbed laser fluence of at least 4 mJ/cm^2^. However, the temporal contrast is reduced to C_0_ = 4 and C_∞_ = 1.5 and the peak reflectivity to 2.4%. When designing a faster switch, one has to make a trade-off between the speed of the temporal response and the diffraction efficiency.

Considering these limiting factors while aiming to speed up the switch response, one could consider utilizing higher cancellation modes at larger energy offsets. Thus, one needs to increase the amount of strain by increasing the laser fluence. However, for a device put to practical use, it is important that even if laser the spot profile changes and pulse-to-pulse intensity fluctuates, the maximum fluence should remain well below the damage threshold. Under the conditions demonstrated here, the gold film did not degrade even though experiments were carried out for several days. Whereas it is possible to theoretically double the laser fluence, we believe that we are close to the practical limit.

## CONCLUSION

In conclusion, we have designed a gold/InSb X-ray switch and characterized the performance. This switch can reduce the pulse duration of the 100 ps X-ray pulse to 20 ps with a contrast of C_0_ = 9, C_∞_ = 4, and a peak reflectivity of 8%. Based on another concept, the PicoSwitch^11^ can reduce the pulse duration to 2 ps with a contrast of C_0_ = 50, C_∞_ = 7 however at the expense of the peak reflectivity of 0.1%. For studies that require high throughput and can afford a temporal resolution of 20 ps, we believe that the gold/InSb switch can find application. It represents a simple way of increasing the temporal resolution of synchrotron beamlines. This study was performed at 2 Hz limited by the LINAC repetition rate. However, time-resolved experiments at storage rings are usually operated in the multi-kHz to MHz range. To implement the switch at those repetition rates, one has to make sure that the laser system can deliver an incident uniform fluence of 50–60 mJ/cm^2^ over an area larger than the X-ray spot size. This requirement excludes the possibility to use MHz repetition rates at present. The switch can be implemented in a double InSb crystal monochromator by coating one of the monochromator crystals with a thin layer of gold. By exciting this gold film with the same laser as is used in the experiment, the new short pulse will be synchronized to the experimental laser.
